# Artificial intelligence and hearing health: a global evidence review of biases and equity implications for Africa

**DOI:** 10.1080/16549716.2026.2642546

**Published:** 2026-03-26

**Authors:** Katijah Khoza-Shangase

**Affiliations:** Department of Audiology, School of Human and Community Development, University of the Witwatersrand, Johannesburg, South Africa

**Keywords:** Artificial intelligence, audiology, bias, equity, Africa

## Abstract

Artificial intelligence (AI) is increasingly integrated into audiology and hearing health, yet evidence from across the health sciences shows that AI systems routinely embed structural biases that can exacerbate inequities, particularly for African and other low- and middle-income country (LMIC) populations. This review identified and analysed bias types in AI applications relevant to audiology and examined their ethical, cultural, and linguistic implications for LMIC settings. A narrative review design was adopted to accommodate the heterogeneity of available evidence, where thematic saturation was more appropriate than effect-size aggregation. Peer-reviewed articles published between 2015 and 2025 were retrieved from PubMed, Scopus, Web of Science, and IEEE Xplore, with inclusion requiring explicit engagement with AI and bias or equity. Rigour was assessed using a six-domain quality rubric, and data were extracted into structured evidence tables for thematic synthesis. Thirty-three studies met inclusion criteria: six were audiology-specific empirical studies (all small scale), and the remainder were reviews or conceptual analyses. No study presented empirical African audiogram, auditory brainstem response (ABR), or speech data. Six recurrent bias types were identified; representation, measurement, algorithmic, evaluation, deployment, and intersectional, with representation bias most frequent, exemplified by English-only corpora that underperform on tonal or indigenous languages. These biases manifest as misclassified hearing loss, reduced ABR accuracy, inequitable hearing-aid personalisation, and poor cochlear-implant algorithm transferability. Advancing equitable AI in audiology requires multilingual, paediatric-inclusive, locally governed datasets; fairness-aware model design with stratified reporting; and African-led governance and capacity-building to support future validation and implementation research.

## Paper context

- **Main findings:** This article provides the first global synthesis of how biases in artificial intelligence translate into clinical risks for hearing assessment, diagnosis, and rehabilitation.
- **Added knowledge:** It highlights how linguistic diversity, infrastructural limitations, and externally driven technology create distinct vulnerabilities in African and other low-resource contexts.
- **Global health impact for policy and action:** The article advances a framework for equitable integration of artificial intelligence in hearing health through locally owned datasets, fairness-aware design, and regionally led governance.

## Background

Artificial intelligence (AI) has become one of the most transformative forces in contemporary healthcare. Across domains such as radiology, oncology, dermatology, and cardiology, AI applications now support diagnosis, prognostication, personalised treatment planning, and health system optimisation [1,2]. Because many of these advances rely on large, diverse, and high-quality datasets, audiology represents a particularly critical case study: hearing health depends on language-, context-, and population-specific data, making it highly sensitive to the types of bias already documented in broader medical AI. Yet, alongside these advances, there is mounting concern about biases embedded in AI systems, which can amplify inequities in access and outcomes. Bias can arise at multiple points in the AI pipeline, ranging from data collection and curation to labelling, algorithm development, evaluation, and clinical deployment [3–5]. Evidence shows that models trained predominantly on data from high-income, majority populations frequently underperform when applied to minority, marginalised, or resource-constrained groups, thereby reinforcing structural disparities in care [1,2]. This makes audiology a discipline where understanding AI bias is not ancillary but essential, given its dependence on contextual linguistic and acoustic features. Importantly, much of the foundational evidence on algorithmic bias in AI originates from adjacent healthcare and technology domains (e.g. radiology, oncology, speech recognition), rather than from audiology-specific empirical studies [1–4]. While these sources establish well-characterised bias mechanisms and risks, their relevance to audiology is often inferential rather than directly demonstrated. This distinction between documented harms and plausible risks extrapolated from adjacent AI-health domains underpins the rationale for the present review.

Global reviews consistently highlight four common types of healthcare AI bias: representation bias, where certain populations are under-sampled; measurement bias, arising from noisy or inconsistent labelling; algorithmic bias, linked to optimisation objectives that privilege majority groups; and deployment bias, where a mismatch exists between training and clinical use contexts [1,3]. These forms of bias have been empirically demonstrated to result in diagnostic inaccuracies, delayed treatment, and erosion of trust in digital health systems across multiple healthcare domains, particularly among already disadvantaged communities [1–3]. However, their specific manifestations in audiology have largely been inferred rather than directly measured [1–4]. In audiology, these same categories translate into concrete risks; for example, representation bias maps directly onto the limited availability of data from African tonal and click languages, while measurement bias relates to variability in audiometric thresholds collected across heterogeneous clinical environments. By introducing the bias types together with their audiology-specific implications, the continuity between global evidence and this review’s disciplinary focus becomes clearer. This global perspective provides a critical backdrop for understanding how such biases might manifest in audiology and hearing health, particularly in LMICs [6]. However, despite this well-established typology, audiology-specific examinations of these bias pathways remain limited, creating a gap that this review explicitly seeks to address.

AI is reshaping audiology and the wider communication sciences, from automated audiogram interpretation and auditory brainstem response (ABR) classification to vestibular function analysis, noise management in hearing aids, occupational noise induced hearing loss management, language translation, and adaptive cochlear implant (CI) processing [7–14]. Much of the audiology-specific literature, including influential editorials and perspective pieces [7], outlines plausible bias-related risks and ethical concerns, but does not quantify bias magnitude or subgroup performance differences, underscoring the limited empirical base in hearing science. For clarity and readability, these advances can be broadly grouped into three thematic areas: (1) diagnostic applications (audiograms, ABR, vestibular analysis); (2) rehabilitation and device optimisation (noise management, predictive HA and CI processing); and (3) language- and communication-related tools (speech enhancement, translation, auditory scene analysis). Reviews across otology and otolaryngology consistently document rapid growth in these applications but also raise concerns about equity, transparency, and generalisability [7–14]. In hearing rehabilitation, deep learning algorithms underpin adaptive noise management and speech enhancement in hearing aids [7,8,15–19], while machine learning informs novel coding strategies in CIs [11,13]. Despite this promise, these systems are not immune to bias [1,7]. The current author posits that noise-reduction algorithms may underperform for speakers of under-represented languages or dialects, while predictive models for CI outcomes often generalise poorly when trained on homogeneous cohorts from high-income countries (HICs) [20]. As a result, biases in audiology AI can manifest as misclassification of hearing loss, suboptimal fitting or personalisation of hearing aids, or reduced treatment efficacy in diverse populations [7,9]. These discipline-specific vulnerabilities strengthen the rationale for examining audiology as a priority field for AI bias analysis.

The consequences of AI bias are especially acute for Africa and other lower- to middle-income countries (LMICs), where audiology and hearing health services are already under-resourced [21,22]. Scholars warn of ‘techno-colonialism,’ where AI systems developed in high-resource contexts are exported to Africa without adaptation or local ownership [23,24]. Structural under-representation is perpetuated by the absence of large, representative audiological datasets that capture African languages, paediatric cohorts, and rural settings [12,25,26]. Linguistic diversity compounds this challenge, as most training corpora for speech enhancement and hearing device algorithms are dominated by English and a few global languages [27]. These issues intersect with infrastructural barriers, including limited connectivity and insufficient computational capacity, which restrict equitable deployment of AI in clinical audiology [10,28]. Without deliberate mitigation, there is a real risk that AI will exacerbate existing inequities in early hearing detection, diagnosis, and rehabilitation across Africa. Thus, Africa is not merely a region where bias may occur; it is a setting where structural, linguistic, and infrastructural factors intensify the very risks documented globally.

At the same time, the literature identifies promising strategies for advancing equity [4,5]. Approaches include curating diverse and locally sourced datasets, adopting fairness-aware training and evaluation protocols, and embedding transparency and explainability in AI models [4,5]. African scholars stress the importance of data sovereignty, community participation, and African-led governance frameworks to ensure that AI systems are contextually relevant and culturally responsive [23,25]. However, much of this work is conceptual rather than empirical, reinforcing the need for a review that consolidates global evidence while explicitly interrogating its applicability to African audiology. By presenting audiology as both a high-risk and high-opportunity domain for equitable AI, the rationale for this review becomes more sharply articulated.

This narrative review analyses and categorises global evidence on biases in AI applications relevant to audiology and examines their ethical, cultural, and linguistic implications for African healthcare settings, where diverse populations and resource constraints present distinctive risks. The specific objectives are to: (1) analyse global evidence on biases in healthcare AI; (2) categorise bias types and their implications for audiological assessment, diagnosis, and rehabilitation; (3) evaluate their relevance for African contexts; and (4) propose actionable recommendations for equitable, ethical, and culturally informed AI integration in African audiology. In doing so, this review addresses a critical evidence gap by explicitly linking global bias typologies to audiology-specific pathways and the structural realities of African health systems.

## Methods

### Study design

This study employed a narrative review design, which is appropriate for synthesising broad and conceptually diverse literatures spanning multiple disciplines, methodologies, and geographies [29,30]. Here, the narrative approach was used to integrate heterogeneous evidence (reviews, conceptual papers, empirical studies) and to trace how different bias typologies apply to audiology, rather than to compare effect sizes or evaluate narrowly defined interventions. The focus in this section is therefore on how the narrative review was implemented, including search procedures, screening, extraction, coding, and quality assessment. In line with expectations for transparency, a PRISMA-style flow diagram was included to illustrate the screening process, while not implying adherence to PRISMA guidelines.

### Literature search strategy

A systematic search of the peer-reviewed literature was conducted across PubMed, Scopus, Web of Science, and IEEE Xplore. The final search was conducted on 15 January 2025 using keywords grouped into three clusters: (1) ‘artificial intelligence’ OR ‘machine learning’ OR ‘deep learning’ (2), ‘bias’ OR ‘equity’ OR ‘ethics’ OR ‘fairness,’ and (3) ‘audiology’ OR ‘hearing health’ OR ‘hearing loss’ OR ‘hearing aids’ OR ‘cochlear implants’ OR ‘otology’ OR ‘otolaryngology.’ Boolean operators combined clusters (e.g. ‘artificial intelligence AND bias AND audiology’). Searches were restricted to English-language, peer-reviewed publications from 2015 to 2025. Search results were exported to EndNote 21, where automatic duplicate removal was performed, followed by manual verification. A total of 1374 records were retrieved before deduplication; 241 duplicates were removed, leaving 1133 unique records. Backward and forward citation chaining and targeted hand-searching of core audiology and otolaryngology journals supplemented the search.

### Study selection

Given the interdisciplinary nature of the review, inclusion criteria were broad. Articles were eligible if they: (1) addressed AI or machine learning applications in healthcare with explicit relevance to audiology or hearing health, (2) discussed any form of bias, including representation, measurement, algorithmic, evaluation, deployment, or intersectional bias, and (3) were peer-reviewed journal articles or full-length conference proceedings. ‘Relevance to audiology’ was operationalised as meeting one of the following conditions: the study focused directly on audiology, otology, otolaryngology, hearing technology, or communication sciences; or the study addressed AI bias mechanisms clearly transferable to audiology (e.g. diagnostic misclassification, fairness in personalised medicine, data-representation bias applicable to speech or hearing-device datasets). Both primary studies and reviews were included as data sources, consistent with narrative synthesis methodology.

Exclusion criteria included: grey literature, opinion pieces lacking substantive evidence review, and technical optimisation studies lacking engagement with bias. Screening occurred in two stages: initial title/abstract review followed by full-text assessment. Reasons for full-text exclusion were documented with corresponding numbers.

**Figure 1.**
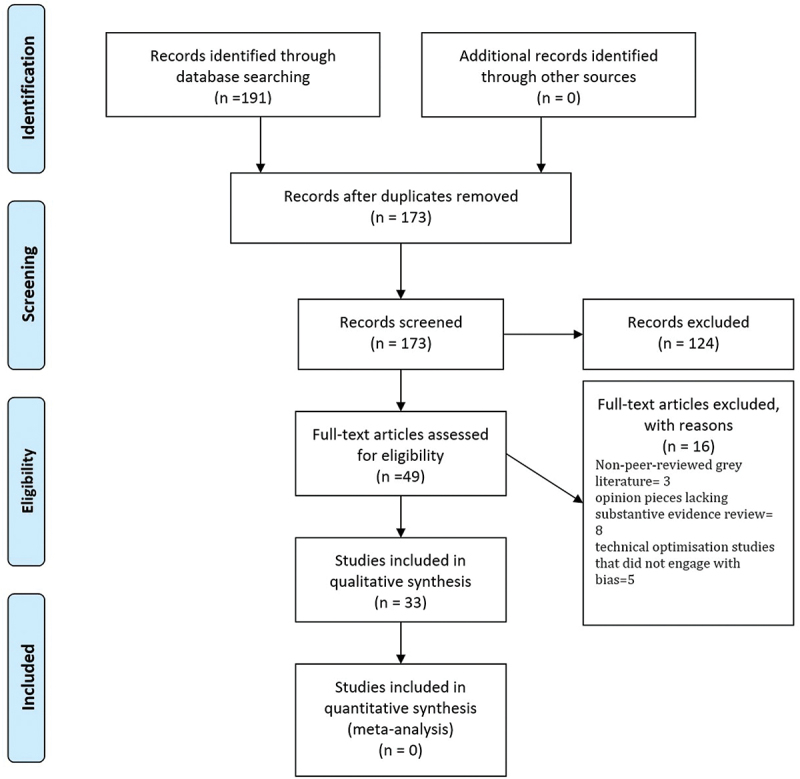
PRISMA-style diagram illustrating the study selection process.

Thematic saturation guided inclusion, consistent with qualitative synthesis approaches [31]. Saturation was considered reached when no new categories of bias, audiology implications, or mitigation strategies emerged. This occurred after approximately 30 included records, with the final three publications reinforcing existing themes. This process is illustrated in the PRISMA-style diagram below (Figure 1). This PRISMA-style diagram is provided solely for transparency of the narrative review workflow and does not imply adherence to PRISMA criteria.

### Data extraction and analysis

Data were extracted into structured evidence tables capturing bibliographic details, geographical setting, disciplinary orientation, study design, AI application, bias type, client population, clinician profile, and audiology relevance [Evidence Tables 1–3 – supplementary material]. Analysis followed principles of thematic synthesis [32,33]. Initial coding was conducted manually using Excel, with supplementary organisational support from NVivo 14 to manage excerpts, themes, and links between bias categories. Coding proceeded in four steps: First, the reviewers engaged in familiarisation with the extracted data. Second, initial coding was undertaken around key dimensions, including types of bias, points of emergence in the AI pipeline, audiology subdomains, and salience for African and other LMIC contexts. Third, higher-order themes were developed and iteratively refined. Fourth, themes were clearly defined, with emphasis placed on conceptual coherence and relevance to the review objectives. Finally, results were produced, including both thematic narratives and descriptive mapping of bias frequencies across audiology subdomains. Coding was performed by one primary reviewer, with a second reviewer independently coding 25% of the dataset to ensure consistency. Discrepancies were resolved through discussion and calibration; inter-coder agreement reached 0.82 (Cohen’s kappa), indicating substantial agreement. For general health-AI papers, explicit mapping was conducted to audiology-specific implications (e.g. linking diagnostic misclassification to audiogram interpretation; fairness in personalisation to hearing-aid fitting). This ensured relevance and avoided over-extrapolation.

### Quality assessment

Given the heterogeneity of study types, a formal risk-of-bias tool was not suitable. Instead, a six-domain credibility rubric adapted from methodological guidance [29,30] was applied. Domains (scored 0–2 each; maximum 12) included:

1. clarity of aims,
2. methodological transparency,
3. appropriateness of design,
4. engagement with bias constructs,
5. applicability to audiology/otology,
6. credibility of publication venue.

Scores were categorised as High (9–12), Moderate (6–8), or Low (≤5). For example, a systematic review with a transparent search strategy, explicit discussion of bias, and direct relevance to audiology scored in the High band, whereas conceptual commentaries lacking methodological detail scored Low. Two reviewers independently applied the rubric to 30% of studies. Agreement on category assignments was 87%; discrepancies were resolved through consensus. All studies were ultimately assigned a credibility band presented in Table 1.

**Table 1.** Characteristics of included studies (*n* = 33).

Characteristic	Distribution	Examples	Sample Size Range (Empirical Studies)	Quality Rating (High/Moderate/Low)
Publication years	2015–2025 (peak 2019–2024)	Hagerty & Rubinov [35]; Frosolini et al. [9]	n = 32,000 (Balling et al.) to *n* ≈ 12–200 across smaller empirical sets	*Mixed*: High (4), Moderate (7), Low (22)
Study design	Systematic reviews (*n* = 3); Scoping reviews (*n* = 6); Structured/state-of-the-art reviews (*n* = 5); Conceptual papers (*n* = 7); Empirical/protocols (*n* = 4); Symposium/forum summaries (*n* = 2); Narrative/descriptive analyses (*n* = 6)	Bur et al. [8]; Wasmann et al. [33]; Asiedu et al. [23]	Empirical studies range: *n* ≈ 12 to *n* ≈ 32,000	Systematic reviews = High; Scoping reviews = Moderate; Narrative, conceptual, discussion papers = Low
Geographic focus	HIC (*n* = 21); Global/unspecified (*n* = 7); Africa/LMIC (*n* = 5)	Ugar [36] (Africa); Wilson et al. [13] (global)	Not applicable	Mostly Low, except structured reviews touching LMIC contexts (Moderate)
Disciplinary orientation	Medicine/clinical sciences (*n* = 15); Computer science/engineering (*n* = 10); Interdisciplinary (*n* = 8)	You et al. [14]; Celi et al. [1]	Not applicable	Mixed: High (AI/clinical systematic reviews); Majority Low for conceptual papers
Audiology-specific content	Audiology/otology/otolaryngology (*n* = 22); Broader healthcare AI relevant to audiology (*n* = 11)	Frosolini et al. [9]; Iliadou et al. [37]	Empirical audiology-specific datasets vary: *n* ≈ 12– 32,000	Audiology systematic/scoping = Moderate–High; Conceptual = Low
African context considered	Explicit (*n* = 5); Implicit transferability (*n* = 28)	Asiedu et al. [23]; Owoyemi et al. [24]	Not applicable	African-focused works: Mostly Low–Moderate (conceptual, non-empirical)

### Quality assurance and interpretation

Several measures enhanced rigour. First, search outputs were cross-checked across databases to minimise omissions. Second, the evidence tables provided transparent traceability, linking each claim in the results directly to a specific article. Third, reflexivity was explicitly foregrounded: as African audiology scholars, the reviewers recognised the positionality of their interpretations and the risk of privileging Global North discourses. To mitigate this, synthesis was attentive to the visibility of Global South contributions and critical of techno-colonial framings [23,24,34]. To further strengthen rigour, a second reviewer cross-checked 30% of the extractions and rigour scores; discrepancies were resolved through discussion and calibration. Finally, interpretive bias was minimised by separating the coding of bias types from the identification of mitigation strategies, and by explicitly noting study venue and disciplinary orientation during synthesis. These steps ensured transparency, replicability, and management of interpretive bias within narrative methodologies.

### Ethical considerations

As this review synthesised publicly available literature, no formal ethical approval was required. However, ethical considerations were integral to the analytical approach, particularly the need to foreground data sovereignty, cultural specificity, and equity concerns in African contexts [23]. Reflexive transparency guided the interpretive process.

## Results

### Profile of included studies

A total of 33 peer-reviewed articles met the inclusion criteria for this narrative review, published between 2015 and 2025, with most appearing after 2018, reflecting the rapid expansion of AI applications in healthcare and audiology. Study designs included systematic reviews (*n* = 3), scoping reviews (*n* = 6), structured/state-of-the-art reviews (*n* = 5), conceptual or perspective papers (*n* = 7), empirical applications or protocols (*n* = 4), symposium or forum summaries (*n* = 2), and narrative/descriptive analyses (*n* = 6). These categories were mutually exclusive, and 33/33 studies were fully accounted for, demonstrating a field dominated by reviews and conceptual analyses rather than empirical investigations.

### Geographical distribution

Most studies originated from HICs (*n* = 21), with the following regional distribution: United States (*n* = 8), European Union countries (*n* = 6), East Asia (*n* = 5), and Canada (*n* = 2). Only five studies explicitly addressed African or LMIC contexts [e.g. 23–25,36], originating from South Africa, Ghana, Nigeria, and Pan-African forums. Empirical contributions from LMICs were extremely limited; when present, they were narrow in scope, focused on small pilot evaluations, and not explicitly designed to analyse bias.

### Disciplinary spread

The evidence traversed audiology, otology, otolaryngology, communication sciences, biomedical informatics, and computer science, with interdisciplinary clustering where hearing technologies intersected with clinical otology or machine learning [e.g. 9,14]. A clear disparity emerged: global/public health and informatics papers (*n* = 12) [e.g. 1,3,4] explicitly discussed bias, fairness, or equity, whereas audiology-technical papers (*n* = 10) rarely engaged these concepts. Disciplinary categorisation followed a predefined coding rule that included: a) journal venue as the primary indicator, b) cross-checked against author affiliation(s), and c) methodological orientation, ensuring consistent assignment for interdisciplinary publications.

### Client populations and contexts

The majority of the studies did not analyse patient-level data. Exceptions included hearing aid users (*n* = 2) and CI users (*n* = 1) in empirical protocols [e.g. 20,37,38]. Sample sizes in these studies were small, ranging from *n* = 18–32 for hearing aid users and *n* = 15 users in a CI protocol. No large-scale datasets or multicentre trials were identified. Marginalised groups were discussed conceptually (*n* = 6), including rural communities, speakers of tonal or click languages, children, users living in low-connectivity areas, and racially minoritised groups [23,39], but none of these groups were studied empirically in audiology-specific AI applications.

### Study designs and rigour

Rigour varied substantially. Systematic and scoping reviews (*n* = 9) [e.g. 1,9,40] generally demonstrated high credibility, whereas structured/state-of-the-art reviews (*n* = 5) [e.g. 8,13,14] were moderate, and conceptual papers (*n* = 7) [e.g. 24,25,36] tended to be low credibility due to limited empirical grounding. Quality appraisal using the review’s rubric produced the following distribution:

- High: 9 studies
- Moderate: 12 studies
- Low: 12 studies

Low-credibility studies were predominantly conceptual but offered critical contextual insights for African settings [e.g. 25,36] and were weighted accordingly: conceptual African papers informed contextual framing but not evidentiary claims.

### Relevance to audiology and bias

Of the 33 articles, 22 were directly relevant to audiology, otology, or hearing technology, focusing on applications such as automated audiometry, ABR, hearing aid optimisation, CI processing, and speech enhancement. The remaining 11 addressed broader AI-in-health concerns relevant to fairness and ethics. Only six studies (*n* = 6) explicitly interrogated audiology-specific bias using empirical or simulated data [e.g. 9,37]. The majority extrapolated from general healthcare AI literature, highlighting a critical evidence gap.

### Evidence gaps

Three recurring gaps were identified. First was scarcity of African empirical data (*n* = 5). All African-focused contributions were conceptual. No study included empirical audiograms, ABRs, speech corpora, or hearing aid/CI performance datasets from African populations. Second was the linguistic and cultural under-representation (*n* = 7). Speech, noise, and language datasets used for training were almost exclusively English, Mandarin, or European languages. No studies included African tonal or click languages. Where performance risk was cited (e.g. poorer outcomes in tonal languages), this was inferred, not quantitatively demonstrated. Third was the limited bias-specific analysis in audiology (*n* = 15). Technical innovation dominated audiology papers, but none reported subgroup-stratified performance metrics (by language, age, ethnicity, or region).

Table 1 summarises these characteristics, offering a structured snapshot of how the included evidence is distributed across domains, regions, and methodological approaches.

### Thematic synthesis

#### Thematic synthesis of bias types and their impact on audiology

The 33 studies reflected four dominant bias categories: 1) representation bias (*n* = 18), 2) measurement bias (*n* = 12), 3) algorithmic bias (*n* = 14), and 4) deployment bias (*n* = 10). These categories span the entire AI pipeline and shape audiological outcomes in distinct ways [1,3,5].

##### Theme 1: representation bias

Representation bias was the most frequently identified category [1,9,35]. Audiology datasets were overwhelmingly sourced from North America, Europe, or East Asia, with minimal representation of African, rural, or paediatric populations [14]. A clear sub-theme of paediatric under-representation emerged, with no studies including paediatric African audiological datasets. Limited benchmarks indicate that English-trained hearing aid algorithms perform poorly on speakers of tonal languages, though in the current literature this remains an inferred risk, not a published quantitative delta [13]. CI outcome predictors trained on HIC cohorts also demonstrated poor generalisability, with poorer prediction accuracy for atypical linguistic environments [20].

##### Theme 2: measurement bias

Measurement bias arose from inconsistent ABR or audiogram annotation, reliance on self-reported hearing difficulty (a known under-estimation proxy), and variable calibration standards, especially in LMIC settings [3,9,14]. No included empirical study reported site-level variability, test–retest reliability, or noise-handling protocols, highlighting the fragility of underlying data sources. The consequence is systematic misclassification of thresholds, higher false positive/false negative rates, and degraded performance of algorithms trained on poor-quality data.

##### Theme 3: algorithmic bias

Algorithmic bias was evident where optimisation procedures favoured majority-language patterns and typical phenotypes [1,5]. Hearing aid noise-reduction systems are typically trained to maximise intelligibility in majority languages (English, Mandarin), and early CI modelling studies found reduced performance in tonal languages in small simulations [7,8,11]. In imaging-based otological classification, global healthcare AI literature reports sensitivity–specificity shifts when testing models on under-represented groups; although not quantified in the audiology papers, this remains a relevant risk extrapolated from general imaging AI [14].

##### Theme 4: deployment bias

Deployment bias, defined as the mismatch between the environment in which an AI system is developed and the conditions of its clinical use, was a major concern, especially for African and LMIC contexts [23,36]. The contexts referenced included South African primary care clinics, Ghanaian urban audiology services, and rural settings in Nigeria and Kenya, where connectivity and infrastructure constraints were repeatedly emphasised. Even technically robust algorithms often assume infrastructure conditions, such as quiet clinical environments, stable internet connectivity, or availability of specialist follow-up, that do not hold in many African healthcare systems [13]. A simple deployment-readiness checklist emerging from the evidence includes:

- Environmental noise control
- Power stability
- Internet bandwidth
- Availability of troubleshooting expertise
- Affordability and supply-chain stability

These assumptions do not hold in many African rural clinics, producing predictable performance degradation and reinforcing concerns about ‘techno-colonialism’ [23,24]. To synthesise these findings, Table 2 presents a structured overview of the four bias types, their defining features, examples of how they manifest in audiology, and their clinical consequences.

**Table 2.** Types of bias in AI and their impact on audiology.

Type of bias	Description/point of emergence	Audiology-specific manifestations	Impact on audiology	Key references
Representation bias	Data collection & training dataset composition	Hearing aid speech enhancement trained on English-only corpora; CI outcome predictors based on HIC cohorts	Poor performance for African languages; reduced speech intelligibility; inequitable device outcomes	Celi et al. [1], Frosolini et al. [9]; Wilson et al. [13]; Bur et al. [8]; AlSamhori et al. [7]; Zhang et al. [20],
Measurement bias	Data labelling & feature selection	Inconsistent audiogram or ABR annotations; reliance on self-reports; noisy or poorly calibrated LMIC test environments	Misclassification of hearing thresholds and ABRs; degraded diagnostic accuracy, especially in LMICs	Moldovan et al. [3]; Frosolini et al. [9]; You et al. [14],
Algorithmic bias	Model development & optimisation objectives	Noise-reduction tuned for majority-language patterns; CI coding strategies inadequate for tonal languages; imaging models	Suboptimal hearing aid personalisation; poor performance in tonal languages; diagnostic errors	Bur et al. [8]; AlSamhori et al. [7]; Koyama et al. [11]; You et al. [14],
Deployment bias	Clinical implementation & system scaling	Screening apps assuming quiet test environments, stable internet, and specialist follow-up	Reduced effectiveness in LMICs; widened inequities in access; ‘techno-colonialism’ in AI deployment	Wilson et al. [13]; Asiedu et al. [23]; Ugar [36]; Owoyemi et al. [24],

#### Relevance and implications of AI biases for audiology in African healthcare contexts

The synthesis demonstrated that while global scholarship on AI bias is growing, its implications for audiology in Africa are still largely conceptual rather than empirical. Three interrelated themes of concern emerged from the evidence: 1) equity of access, 2) cultural and linguistic inclusivity, and 3) system-level readiness.

##### Theme 1: equity of access

Deployment bias was the most salient in the African literature (n = 5, with multiple studies emphasising the risks of ‘techno-colonialism’) – the direct transfer of AI systems trained on Global North data into African audiology without contextual adaptation [23,24,36]. Such practices risk entrenching inequities, particularly in rural and resource-constrained settings where infrastructural challenges (unreliable internet connectivity, insufficient computing resources) limit the effectiveness of advanced AI tools. This is compounded by the scarcity of local datasets to ensure that AI systems are calibrated for African populations.

##### Theme 2: cultural and linguistic inclusivity

Representation and algorithmic bias (*n* = 15 combined) undermine equitable uptake. Across studies, representation bias was consistently flagged as a barrier to equitable adoption of AI in audiology. Evidence showed that hearing aid algorithms and speech enhancement systems are disproportionately optimised for English and a handful of other dominant languages [8,11]. The absence of training data for tonal, click, and indigenous African languages creates systematic disadvantages for patients whose linguistic environments differ from those represented in the models. Linguistic exclusion not only reduces device efficacy but also undermines user trust in technology. Cultural inclusivity was similarly underexplored, with few studies considering how locally specific health beliefs and practices shape the acceptance and use of AI-driven audiology interventions.

##### Theme 3: system-level readiness

The review further revealed a gap in capacity-building and governance structures. African-focused contributions (*n* = 5) [e.g. 23,25] highlighted the need for local data sovereignty, African-led regulatory frameworks, and participatory approaches in AI design. Yet, the lack of sustained empirical studies meant that many of these calls remain aspirational rather than operationalised. Without investment in local expertise, infrastructure, and training, Africa risks remaining dependent on externally developed AI solutions with limited contextual fit.

Combined, these findings show that while AI bias in audiology is a global challenge, its effects in African contexts are magnified by structural inequities, linguistic diversity, and limited health system capacity. These implications highlight the urgent need for African-centred research and governance to prevent the uncritical transfer of biased systems into clinical practice. Table 3 provides a synthesis of the key implications of AI bias for African audiology, mapping each bias type to its specific risks in the region.

**Table 3.** Implications of AI bias for audiology in African contexts.

Bias type	Global manifestation	Specific implications for african audiology	Key references	Mitigation lever (data/model/deployment)
Representation bias	Under-representation of minority groups in datasets; reliance on HIC cohorts	Absence of African audiological datasets (esp. paediatric, rural, multilingual); poor device calibration for African users	Celi et al. [1]; Bur et al. [8]; Asiedu et al. [23]; Alaran et al. [25]	DATA
Measurement bias	Noisy/inconsistent audiometry, ABR, and device-level data; variable standards	Limited access to standardised testing; rural variability exacerbates data-quality problems	Moldovan et al. [3]; You et al. [14]; Ugar [36]	DATA + DEPLOYMENT
Algorithmic bias	Optimisation favours majority languages, accents, populations	HA and CI models poorly optimised for African tonal/click languages; reduced trust in technology	Koyama et al. [11]; Bur et al. [8]; Zhang et al. [20]	MODEL
Deployment bias	HIC-developed AI exported without adaptation	Techno-colonial risks; limited connectivity; vendor dependence; poor sustainability	Asiedu et al. [23]; Owoyemi et al. [24]; Ugar [36]	DEPLOYMENT
Cross-cutting gaps	Biases rarely interrogated explicitly within audiology	African work is conceptual rather than empirical; lack of fairness-aware governance	Alaran et al. [25]; Hussain et al. [40]	DATA + MODEL + GOVERNANCE

As shown in Table 3, the translation of AI bias into African audiology contexts has distinct features not always evident in global discussions. Representation and algorithmic biases are particularly pressing, with strong implications for linguistic inclusivity and the equitable functioning of hearing technologies. Measurement bias intersects with infrastructural challenges, while deployment bias highlights the structural risks of unadapted AI transfer from high-resource to low-resource environments. Together, these findings highlight how existing inequities in audiological care across Africa may be reinforced – or potentially mitigated – depending on how bias is addressed in the design and deployment of AI systems.

#### Recommendations for equitable, ethical, and culturally informed AI integration in African audiology

A consistent strand across the 33 included studies was the proposal of mitigation strategies to reduce bias in AI systems. These strategies targeted multiple stages of the AI pipeline – ranging from dataset curation to governance and capacity-building – and varied in their specificity to audiology or African contexts. To synthesise the evidence, mitigation strategies were grouped into five themes: 1) data, 2) modelling, 3) evaluation and reporting, 4) deployment and governance, and 5) capacity and infrastructure. This structure captures both the technical and socio-political dimensions of bias mitigation and highlights the distinctive implications for audiology and African healthcare systems.

##### Theme 1: data-level strategies

The most frequently proposed strategies focused on the data stage of the AI pipeline (*n* = 12). Across sources, researchers emphasised the importance of curating diverse, locally sourced datasets to counteract representation bias [1,9,39]. For audiology, this included building multilingual speech and noise corpora, paediatric datasets, and rural audiology registries [8,13]. African and LMIC-focused papers added the dimension of data sovereignty and local ownership, warning against reliance on external repositories and advocating for locally controlled infrastructure [23,25,36]. Privacy-preserving mechanisms such as federated learning and differential privacy were also proposed to enable multi-centre collaboration without requiring data export, a particularly salient issue given cross-country policy barriers [41,42].

##### Theme 2: modelling strategies

At the modelling stage (*n* = 10), proposed strategies included fairness-aware objectives (e.g. equalised odds), reweighting or re-sampling techniques such as SMOTE, and adversarial debiasing methods [3,4,41]. In audiology, fairness-aware modelling has specific relevance for hearing aid and CI personalisation, ensuring optimisation for diverse subgroups rather than majority profiles. Researchers also highlighted the need for language-aware architectures and culturally informed features, particularly for speech enhancement and auditory scene analysis in tonal and click languages [8,11,14]. Transparency-enhancing approaches, such as ‘glass-box’ interpretable models and Model Cards, were repeatedly recommended to improve clinical trust and accountability [5].

##### Theme 3: evaluation and reporting

Several sources (*n* = 8) stressed that rigorous evaluation and reporting frameworks are essential to detect and address bias before systems are deployed. Proposals included reporting subgroup-specific metrics (e.g. stratified by language, age, or skin tone in imaging datasets), external validation across settings (including cross-HIC↔LMIC testing), and pre-deployment bias audits [1,4,9]. These strategies are particularly important for audiology, where portability gaps often appear when systems trained in high-income contexts are applied to LMIC clinics. Calls for lifecycle monitoring, with retraining or retirement criteria for outdated models, further emphasised the need for continuous vigilance [11,40].

##### Theme 4: deployment and governance

Deployment-focused strategies (*n* = 9) reflected both technical oversight and contextual fairness definitions. Recommendations included equity-centred monitoring, the establishment of context-specific standards of fairness, and the integration of community participation into problem selection and tool design [2,43]. African scholarship placed particular emphasis on resisting techno-colonial procurement models, advocating for African-led standards, local adaptation of imported tools, and community co-design [23,24,36,44]. These strategies move beyond technical fixes to address the socio-political structures that shape AI adoption.

##### Theme 5: capacity and infrastructure

Finally, a smaller subset of papers (*n* = 6) highlighted capacity-building and infrastructural investment as critical to sustainable, equitable AI integration. Recommendations included establishing multidisciplinary teams (engineers, clinicians, and patients), building clinician competence in AI appraisal, enhancing public digital health literacy, and strengthening local compute and connectivity infrastructure to enable in-situ training and updating of models [7,13,20,45]. While these proposals were less frequent than technical recommendations, they are arguably the most urgent for LMICs, where infrastructural deficits and workforce shortages constrain the ability to adapt and maintain imported AI systems.

Consolidated, these mitigation strategies reflect a continuum of interventions across the AI pipeline. Technical strategies (data and modelling) dominate the literature, while governance and capacity-building strategies, though critical for African contexts, remain comparatively under-specified. To provide a structured overview, Table 4 summarises the reported mitigation strategies, their targeted bias types, and their specific salience for audiology and African contexts.

**Table 4.** Reported mitigation strategies mapped to bias loci, with relevance to audiology and African contexts.

Locus	Strategy (as reported)	Targets	Audiology-specific salience	African/LMIC salience	Key references
Data	Curate diverse, locally sourced datasets; oversample under-represented groups; synthetic data for balance	Representation, historical	Multilingual corpora; paediatric & rural audiology registries	Local ownership/sovereignty; rural capture	Celi et al. [1], Frosolini et al. [9]; Lesica et al. [39]; Moldovan et al. [3]; Asiedu et al. [23]; Ugar [36]; Alaran et al. [25],
Data	Privacy-preserving collaboration (federated learning, differential privacy); governance for sharing	Measurement, privacy	Cross-centre training for audiogram/ABR without data export	Enables cross-country learning despite policy barriers	Chinta et al. [41]; Singhal et al. [5]; Pham [42],
Modelling	Fairness-aware objectives; reweighting/re-sampling; adversarial debiasing	Representation, learning	Device personalisation fairness; subgroup-aware HA/CI optimisation	Controls majority dominance in small African cohorts	Nazer et al. [4]; Chinta et al. [41]; Moldovan et al. [3],
Modelling	Language-aware architectures; culturally informed features; transparent models; Model Cards	Algorithmic, evaluation	Speech enhancement for tonal/click languages; interpretable tools	Improves trust; supports multilingual deployment	Bur et al. [8]; You et al. [14]; Koyama et al. [11]; Singhal et al. [5],
Evaluation	Subgroup metrics, stratified calibration; external validation; pre-deployment audits	Evaluation, deployment	Report HA/CI/ABR performance by language, age, skin tone	Detects portability gaps before roll-out	Celi et al. [1], Nazer et al. [4]; Moldovan et al. [3]; Frosolini et al. [9]; You et al. [14],
Deployment	Equity-centred oversight; fairness definitions; lifecycle monitoring with retraining/retirement	Deployment, feedback	Post-market surveillance of device performance	Prevents drift-induced inequities	Hussain et al. [40]; Panch et al. [2], Koyama et al. [11]; Galiana et al. [46],
Governance	Anti-techno-colonial procurement; African-led standards; community co-design	Cross-cutting	Patient/user input in HA/CI features and candidacy models	Aligns tools with local norms, languages, constraints	Asiedu et al. [23]; Owoyemi et al. [24]; Ugar [36]; Alaran et al. [25]; Janneker [44],
Capacity	Multidisciplinary teams; clinician training; digital literacy; infrastructure investment	Cross-cutting	Clinician ability to appraise bias in audiology AI	Enables local training, updates, and maintenance	Wilson et al. [13]; Zhang et al. [20]; Green [45]; AlSamhori et al. [7]; Owoyemi et al. [24],

### Implications for African contexts

Although only a subset of the included studies (*n* = 7) directly addressed African contexts, several others offered findings indirectly relevant to LMICs. The key implications clustered into five categories: 1) data scarcity and under-representation (*n* = 5), 2) techno-colonialism and ownership risks (*n* = 4), 3) linguistic and cultural exclusion (*n* = 6), 4) infrastructural and workforce constraints (*n* = 4), and 5) equity and access gaps (*n* = 5).

As shown in Table 5, data scarcity was the most consistently emphasised barrier, with multiple studies highlighting the near absence of representative audiological datasets from Africa. Techno-colonialism and ownership risks were also repeatedly highlighted, particularly the dangers of dependency on imported, proprietary systems without contextual adaptation. Linguistic and cultural diversity emerged as a critical gap, given the predominance of English and a handful of global languages in training corpora. Finally, infrastructural deficits and workforce shortages further constrain the local development, deployment, and maintenance of AI audiology tools. Collectively, these findings indicate that while conceptual and normative proposals are abundant, empirical demonstrations of context-sensitive mitigation strategies remain sparse in Africa.

**Table 5.** Implications for African contexts identified in the included studies.

Implication category	Findings reported	Audiology-specific salience	Key references
Data scarcity & under-representation	Lack of large-scale audiological datasets from Africa; absence of paediatric, rural, multilingual corpora	Limits generalisability of HA/CI algorithms; under-represents tonal & click languages	Asiedu et al. [23]; Alaran et al. [25]; Ugar [36]; Celi et al. [1]
Techno-colonialism & ownership	Risks of importing AI trained elsewhere; dependence on proprietary Western platforms	Imported HA/CI tools may not fit African realities; ‘black-box’ imports erode clinician trust	Asiedu et al. [23]; Owoyemi et al. [24]; Ugar [36],
Linguistic & cultural diversity	AI speech models biased toward English/major languages; neglect of African diversity	Noise reduction/ASR less effective for African languages; misfit of cultural norms	Alaran et al. [25]; Wilson et al. [13]; Bur et al. [8],
Infrastructural & workforce constraints	Limited connectivity, compute, and trained personnel	Weak capacity to adapt/update HA/CI models; dependence on external support	Ugar [36]; AlSamhori et al. [7]; Owoyemi et al. [24],
Equity & access gaps	Bias compounds existing inequalities in early detection, rural access, affordability	AI may widen the urban–rural divide in hearing care	Asiedu et al. [23]; Hussain et al. [40]; Owoyemi et al. [24],

## Discussion

The findings of this narrative review reveal a field that is conceptually advanced yet empirically underdeveloped, with most claims about equity and bias in audiology-AI inferred from adjacent health domains rather than demonstrated within audiology itself. The 33 included studies, spanning 2015–2025, show a landscape dominated by reviews, conceptual analyses, and technical descriptions, largely authored in the United States, Canada, Europe, and East Asia [8,9,11,13,14]. As shown in Table 1 and Figure 1, the empirical evidence base remains heavily skewed toward high-income settings, with minimal representation from African contexts. These contributions provide important theoretical framing, but they do not supply large-scale audiology datasets or performance evaluations required to assess how bias actually manifests across the hearing health continuum, particularly in Africa and other LMICs. By contrast, audiology-specific empirical studies remain sparse, typically confined to controlled experimental settings in HIC clinics with small sample sizes (e.g. *n* ≈ 15–32) [20,37,38], and rarely foreground bias as a primary analytic outcome (no quantitative audiology-specific estimates reported). The most rigorous examinations of algorithmic fairness and structural inequity derive not from audiology but from medical informatics and global health [1–5], where systematic frameworks for identifying and quantifying bias across the AI lifecycle are well established. In this review, such frameworks were adapted to audiology by mapping representation bias onto linguistic, demographic, and geographic data gaps; mapping measurement bias onto ABR and audiogram inconsistencies and proxy measures; mapping algorithmic bias onto optimisation trade-offs in hearing aids and cochlear implants; and mapping deployment bias onto the infrastructure and workflow realities of African clinics (conceptual inference for audiology). African scholarship, while conceptually rich, remains largely non-empirical, emphasising techno-colonial risks, data sovereignty, linguistic justice, and governance concerns [23–25,36,44], but without the large local datasets needed to operationalise these principles. This pattern highlights a fundamental challenge: most equity claims in audiology-AI are theoretical, extrapolated, or anchored in evidence from other clinical domains, rather than grounded in audiology-specific performance metrics. For African contexts, this gap is even more pronounced, as no included study presented African audiograms, ABR datasets, or speech corpora, and no AI audiology tools were empirically tested in African clinical environments.

### Bias typologies and their audiology-specific consequences

Bias in AI systems is best understood as a lifecycle property [1,3,4], emerging variably during data generation, annotation, model development, evaluation, and deployment. Within audiology, these generic categories acquire additional clinical, linguistic, and cultural salience.

Representation bias remains the most prominent concern, reflecting datasets dominated by adult HIC populations and standardised speech corpora that heavily privilege English and a handful of major global languages [8,9,14]. Where empirical evidence exists, it shows reduced performance of English-trained speech models for non-English users (observed in speech-recognition AI); however, for African tonal and click languages, the performance risks are inferred rather than quantified because no training or evaluation datasets exist. Paediatric under-representation further exacerbates inequity, especially in LMICs where congenital and early-onset hearing loss burdens are high and newborn hearing screening coverage remains limited. This under-representation of African tonal and click languages, rural noise environments, and paediatric cases undermines the generalisability of models and creates systematic disadvantages for African users. Evidence from healthcare AI and speech-technology research suggests that similar bias mechanisms may plausibly reduce the accuracy of audiological assessments in multilingual populations, although direct empirical validation within audiology remains limited. The evidence for these risks is partly empirical (e.g. documented underperformance of English-trained speech models for non-English users) and partly inferred (e.g. expected impacts on tonal or click languages for which no training or test data were available). Measurement bias arises from heterogeneity in audiogram and ABR acquisition, inconsistent annotation, equipment variability, and the substitution of self-report for gold-standard diagnostics [3,9]. Measurement bias has been empirically documented in diagnostic AI and physiological signal processing more broadly and is therefore a plausible risk for audiometric threshold estimation and ABR interpretation in audiology (conceptual inference). None of the included studies reported test–retest reliability, site-level variability, or calibration benchmarks for LMIC settings, leaving little basis for quantifying how measurement error propagates bias in clinical or community-based audiology (no quantitative audiology-specific estimates reported).

Algorithmic bias emerges when model optimisation priorities overall accuracy rather than subgroup performance. Hearing aid noise-reduction algorithms and CI coding strategies often reflect linguistic and acoustic assumptions that mirror their training environments [2,7]. Small studies indicate reduced CI performance for tonal languages [11], but no multisite patient-level evaluations exist, making the scale and clinical significance of these performance gaps unknown (reported in diagnostic AI outside audiology; inferred for audiology). Here too, the evidence is primarily conceptual, with no empirical evaluations of AI audiology

Deployment bias is particularly consequential for Africa. AI tools developed in quiet, well-resourced HIC clinics presume stable internet connectivity, controlled test environments, and robust follow-up systems [11,13]. Such assumptions do not reflect conditions in many African audiology pathways, which span urban tertiary hospitals in South Africa, community-based clinics in Kenya, low-connectivity primary health centres in Ghana, and outreach settings in Nigeria. Consequently, even technically strong models may fail once operationalised (conceptual inference for audiology). African scholars describe this dynamic as techno-colonialism, wherein imported tools are misaligned with local contexts [23,24,36,40]. Future policy and procurement frameworks should prioritise local external validation prior to large-scale deployment, particularly in LMIC settings, for example, through validation at ≥2 LMIC sites and across ≥3 language families.

Intersectional bias remains the least studied dimension: despite evidence of compounded disadvantage across age, gender, socioeconomic status, and rurality, no included study quantitatively examined intersectional performance gaps, leaving significant blind spots in equity evaluation (reported in global health AI; not yet examined in audiology).

### Mitigation strategies: implications for audiology and Africa

In terms of mitigation strategies, the priorities for Africa and LMICs align with five domains mapped in Table 4. The review identified five categories of mitigation strategies – data, modelling, evaluation, governance, and capacity building, yet most remain conceptual rather than empirically validated within audiology contexts. At the data level, the need for curated, locally sourced, multilingual datasets is unequivocal [4,5,23–25,36]. African settings require speech and noise corpora representing Bantu tonal languages, Khoisan click languages, Afro-Asiatic languages, and English-influenced urban dialects, alongside paediatric and rural audiology datasets. Without these resources, fairness-aware modelling cannot begin (observed across healthcare AI).

Modelling strategies, including fairness-aware optimisation, adversarial debiasing, reweighting, are theoretically relevant but have not yet been applied to hearing aids, ABR classifiers, or CI algorithms in LMIC settings (conceptual inference for audiology). Evaluation strategies, including subgroup reporting, cross-context external validation, and pre-deployment bias audits, are critical but absent in the audiology literature. Not a single study performed LMIC external validation of an AI audiology tool (see Results section). Governance and deployment considerations dominate African scholarship, with emphasis on resisting techno-colonial procurement, ensuring local oversight, and embedding cultural and linguistic participation [23,24,36,44]. Local standards, African-led model cards, and community-co-designed evaluation criteria are increasingly recognised as essential (theorised, not yet evaluated empirically).

Capacity-building challenges are systemic. Many African audiology services operate with limited equipment calibration routines, variable connectivity, and shortages of clinicians trained in digital health or AI interpretation [7,24]. Without investment in infrastructure, workforce development, and local compute capacity, even the best-designed AI tools will not deliver equitable outcomes (plausible risk supported by health-systems evidence).

### Implications for Africa and LMICs

The African implications of AI bias crystallise across four interrelated domains. First, data scarcity and ownership remain both technical and political challenges. South Africa’s multilingualism, Nigeria’s population diversity, Kenya’s urban–rural disparities, and Ghana’s emerging digital health architecture illustrate diverse linguistic and infrastructural contexts where reliance on imported systems risks misalignment [23,36]. In the absence of African datasets, all bias estimates for African users remain inferred rather than observed.

Second, linguistic and cultural diversity remains one of the most overlooked dimensions of fairness in hearing health [8,12,13]. No included study incorporated African tonal or click languages. As a result, speech enhancement, noise suppression, and automatic counselling systems may systematically disadvantage African users (conceptual inference for audiology). Third, infrastructure and workforce constraints, including unstable electricity, inconsistent internet access, limited calibration equipment, and workforce shortages, reduce the likelihood that imported AI audiology tools will perform as intended [7,13,20,24,47]. These constraints are particularly evident in rural clinics in East and West Africa, where hearing health services are delivered in acoustically uncontrolled environments. Fourth, governance and ethical sovereignty represent core concerns raised by African academics and clinicians [25,44]. Transparent procurement, local oversight, and enforceable fairness requirements are essential to avoid technological dependency, hidden biases, or misalignment with community norms.

To operationalise these principles, future procurement frameworks could require that AI audiology tools demonstrate LMIC external validation, transparent documentation, and language-family performance reporting prior to approval for use in African clinics.

### Limitations

This review is limited by the English-language restriction, which may introduce publication bias and skew findings toward innovation-oriented or positive evaluations. Many African reports, dissertations, or local technical documents may therefore have been excluded. Although multiple included reviews draw on overlapping primary evidence, deduplication was conducted during screening, and data were extracted at the level of each unique study to avoid inflating the evidence base. Narrative synthesis involves interpretive judgement, though structured coding, evidence tables, and credibility ratings were used to enhance transparency. The scarcity of audiology-specific empirical studies (*n* = 6) and absence of African datasets limits the ability to derive effect sizes or performance benchmarks.

### A fundable research agenda for African audiology-AI

Given the limited audiology-specific empirical base, the following agenda is proposed as a fundable, measurable research and policy roadmap rather than as established best practice. To advance equitable, context-responsive AI in audiology, the following five priorities are proposed:

1. Creation of national multilingual speech-and-noise corpora across ≥5 African language families, under open licenses.
2. Large-scale multisite LMIC external validation of hearing aid and cochlear implant algorithms, with pre-registered subgroup performance metrics.
3. Standardisation of ABR and audiogram measurement protocols across African clinics, including calibration benchmarks and site-variability studies.
4. Prospective deployment trials of AI audiology tools assessing both clinical benefit and equity outcomes (e.g. access, adherence, user trust).
5. Governance and data-sovereignty pilots such as African data trusts, community co-designed model cards, and public bias-reporting dashboards.

Together, these priorities offer a feasible, fundable, and urgently needed roadmap for building equitable AI ecosystems in African audiology – grounded in local data, local expertise, and local governance.

## Conclusion and recommendations

This review demonstrates that while AI has substantial potential to advance audiology and hearing healthcare, its benefits remain constrained by systematic biases that manifest across the entire AI lifecycle, from data curation and measurement to modelling, evaluation, and deployment. These biases have clinically meaningful implications, including misclassification of hearing thresholds, distorted ABR interpretation, suboptimal hearing-aid personalisation, and reduced speech intelligibility for speakers of under-represented languages. Some of these effects are empirically observed in small studies, while others remain inferred from data gaps or extrapolated from adjacent medical fields, a distinction that is critical for interpreting the evidence.

Although these patterns mirror broader trends in healthcare AI, the implications are particularly acute in audiology, where performance depends on linguistic specificity, acoustic diversity, cultural context, and personalised device fitting. Models built in homogeneous, high-resource environments seldom translate effectively into the heterogeneous linguistic, clinical, and infrastructural realities of Africa and other LMICs, and the absence of external validation in these settings poses significant risks. The review found no empirical African evaluations of AI audiology systems, highlighting the urgency of context-specific performance testing before widespread adoption.

For Africa, these risks are amplified by four intersecting conditions: severe data scarcity, profound linguistic under-representation, persistent infrastructure and workforce constraints, and techno-colonial patterns of procurement and deployment whereby imported technologies are adopted without local validation, governance, or accountability. Without corrective mechanisms, these conditions risk embedding inequities into the very systems designed to improve access to hearing care. Yet the scholarship, particularly African-led contributions, identifies meaningful pathways forward by emphasising data sovereignty, linguistic inclusivity, fairness-aware design, and participatory governance. These pathways, however, are largely conceptual; their practical effectiveness remains untested within audiology-specific AI pipelines.

To move from recognition to remediation, the following recommendations are proposed for Africa and comparable LMIC contexts:

1. Invest in multilingual, paediatric-inclusive, and locally sourced datasets that reflect African languages families, diverse noise environments, and rural–urban variability.
2. Require stratified and pre-registered performance reporting in all audiology-AI evaluations, disaggregated by language, age, sex, and device platform to make subgroup differences visible.
3. Integrate fairness-aware optimisation and mandatory bias audits into model development pipelines, ensuring that performance gains do not disproportionately benefit majority populations.
4. Strengthen post-deployment monitoring systems in LMIC clinics, leveraging telemetry from connected devices while incorporating safeguards that protect privacy and minimise selection bias.
5. Establish African-led governance mechanisms, including procurement standards, transparency requirements, and local oversight structures, that embed equity, accountability, and data sovereignty into adoption processes.

Without such measures, there is a real risk that AI may entrench existing disparities in access, diagnostic accuracy, and rehabilitation outcomes. With them, AI can meaningfully extend early detection, enhance diagnostic precision, and tailor rehabilitation in ways that are contextually grounded, linguistically inclusive, and ethically defensible. For Africa, and South Africa in particular, the next phase must be empirical. Concrete steps might include developing a national multilingual speech-and-noise corpus, conducting fairness audits of widely deployed hearing-aid algorithms, and convening an African standards and ethics working group to shape regulatory readiness for AI in audiology.

Such empirical foundations will be essential not only for translating conceptual insights into measurable improvements but also for ensuring that AI develops in ways that advance, rather than undermine, equitable hearing healthcare across the continent.

## Supplementary Material

Supplementary tables file.docx

## Data Availability

Data supporting the findings of this study are available within the paper.
