# Supplementary material for: Artificial intelligence and hearing health: a global evidence review of biases and equity implications for Africa
Source: Glob Health Action. 2026 Mar 26;19(1):2642546. doi: 10.1080/16549716.2026.2642546 (PMC13023006; doi:10.1080/16549716.2026.2642546)
Supplement: Supplementary tables file.docx [file ZGHA_A_2642546_SM2152.docx]

**Evidence Table 1:** Study Characteristics

| **Authors/Year of publication** | **Type of Study/Review** | **Main Objective/Purpose** | **Participants/Data Sources** | **Key Methods** |
| --- | --- | --- | --- | --- |
| Asiedu et al. [23] | Scoping review & qualitative study | To explore fairness for global health, with Africa as a case study, proposing axes of disparities and obtaining corroborative evidence. | 672 general population participants; 28 experts in ML, health, and policy (Africa-focused). | Searched peer-reviewed literature to identify axes of disparities; conducted expert surveys, in-depth interviews, and general population surveys. |
| Wilson et al. [13] | Narrative review | To present a wide diversity of applications of AI and ML in otolaryngology and the communication sciences. | Peer-reviewed literature (various databases, 2010–2022). | Reviewed and synthesised literature on AI applications in otolaryngology. |
| Koyama et al. [11] | Narrative review | To discuss ethical, legal, and privacy issues related to AI in clinical medicine, with a focus on otology. | Peer-reviewed literature (2010–2024). | Reviewed and synthesised literature on AI ethics in clinical otology. |
| Lesica et al. [41] | Perspective/Conceptual paper | To discuss challenges in assembling comprehensive datasets for AI in hearing healthcare, particularly in LMICs. | Peer-reviewed literature and conceptual discussion. | Discussed challenges in data availability and efforts to develop datasets for AI. |
| Ferdous et al. [6] | Comprehensive/systematic review | To review the potential of AI-driven Smart Auditory Health Systems (SAHS) in addressing auditory healthcare challenges, particularly in LMICs. | Peer-reviewed literature (PubMed, Scopus, IEEE Xplore, Google Scholar; 2013–2024). | Searched databases systematically; analysed and synthesised findings. |
| Hussain et al. [38] | Scoping review | To examine racial and ethnic bias in AI health algorithms, stakeholder oversight, and consequences for health equity in the US. | 23 peer-reviewed articles and government publications (2020–2024). | Followed PRISMA-ScR; conducted qualitative content analysis. |
| Ismail [16] | Meta-analysis | To analyse the effectiveness of AI-assisted methods in enhancing HA operations and ethical issues in AI adoption. | 10 experimental and comparative studies (2018–2024). | Extracted quantitative data; conducted statistical meta-analysis using SPSS; thematically analysed qualitative data. |
| Fabry & Bhowmik [15] | Conceptual paper | To define human intelligence and AI, and discuss the adoption of AI in hearing devices. | Peer-reviewed literature. | Discussed definitions and applications of AI in HA technologies. |
| Mwangi [17] | Descriptive empirical study | To evaluate the efficacy of AI and advanced noise management in modern HAs for individuals with hearing impairments. | Secondary data (clinical trial datasets, user reports). | Analysed secondary data using descriptive design and regression modelling. |
| Iliadou et al. [37] | Conceptual paper | To present a predictive model for HA users and discuss ethical considerations. | Peer-reviewed models and existing datasets. | Discussed predictive models and their ethical implications. |
| Balling et al. [36] | Empirical research study | To describe collaboration between HA users and AI to optimise sound preferences. | n=32,000 paired A–B comparisons from HA users via smartphone app. | Analysed anonymised preference assessment data with ML techniques. |
| Zhang et al. [20] | Conceptual review | To provide an overview of AI functionalities in hearing care for CIs and HAs. | Peer-reviewed literature (2015–2025). | Discussed AI applications in hearing care technologies. |
| Chinta et al. [42] | Narrative review | To examine AI integration in healthcare, highlighting bias and strategies for mitigation. | Peer-reviewed literature (2010–2024). | Categorised and synthesised biases across the ML pipeline. |
| Moldovan et al. [3] | Systematic review | To investigate how bias manifests across the AI lifecycle in healthcare and its implications. | 97 peer-reviewed articles (6 databases, October 2024 search). | Applied PRISMA 2020; systematically reviewed literature. |
| Pham [43] | Narrative review | To discuss AI advancements in healthcare imaging and ethical, legal, and privacy concerns. | Peer-reviewed literature. | Reviewed and discussed AI in medical imaging and related issues. |
| Green [46] | Conference summary | To summarise proceedings of a conference on AI in CSD, covering clinical care advances. | Conference presentations. | Summarised sessions and thematic discussions. |
| Singhal et al. [5] | Scoping review | To explore ethical use of AI/ML in healthcare information on SMPs, focusing on FATE principles. | 672 records screened (PubMed, Web of Science, Google Scholar). | Followed PRISMA-ScR; charted data on FATE principles. |
| Galiana et al. [44] | Narrative review | To explore ethics and AI in medicine, ensuring safe and fair deployment. | Peer-reviewed literature. | Discussed ethical considerations such as explainability and liability. |
| Owoyemi et al. [24] | Discussion paper | To discuss AI implementation challenges in Africa (data availability, quality, ethics/policy). | Conceptual discussion with African focus. | Discussed challenges and implications for AI in African healthcare. |
| Janneker [45] | Conceptual paper | To address AI bias and ethical challenges in South African healthcare AI. | Conceptual discussion (South African context). | Discussed sources of bias and proposed solutions. |
| Alaran et al. [25] | Discussion paper | To highlight AI’s role in African healthcare provision and policy, addressing ethics. | Conceptual discussion. | Discussed ethical issues including equity, privacy, accountability. |
| Panch et al. [2] | Conceptual viewpoint | To define algorithmic bias in health systems and propose countermeasures. | Conceptual discussion. | Discussed challenges of fairness and black-box algorithms. |
| Nazer et al. [4] | Conceptual paper | To discuss AI bias, sources, and mitigation strategies. | Conceptual discussion based on literature. | Discussed bias identification, data diversification, and model validation. |
| Ugar [35] | Conceptual/Philosophical paper | To argue for African agency and values in AI healthcare design. | Conceptual analysis (SSA healthcare context). | Philosophical argumentation and critique of techno-colonialism. |
| Frosolini et al. [9] | Scoping review | To provide an overview of AI applications in audiology, exploring ethics and clinical implications. | 104 articles (PubMed, Google Scholar; 1990–2024). | Applied PRISMA extension; synthesised AI audiology applications. |
| AlSamhori et al. [7] | Narrative review | To analyse AI applications in audiometry and HAs and associated ethical issues. | Peer-reviewed literature. | Reviewed AI applications in audiometry and HAs. |
| You et al. [14] | Scoping review | To provide an overview of AI applications in otology, including HA optimisation and vestibular disorders. | 38 articles (from 1374 initial hits, MEDLINE & EMBASE). | Conducted scoping review using PRISMA; searched MeSH/keywords. |
| Bur et al. [8] | Narrative review | To discuss origins, definitions, ethical concerns, and practice implications of AI in otolaryngology. | 54 articles screened. | Reviewed and discussed AI in otolaryngology. |
| Celi et al. [1] | Systematic review | To synthesise sources of bias in AI perpetuating healthcare disparities. | Peer-reviewed global health AI studies. | Conducted systematic review and conceptual synthesis. |
| Hagerty & Rubinov [39] | Narrative review | To review global AI ethics and social implications. | Broad ethics and AI governance literature. | Conducted narrative synthesis of ethical/social issues. |
| Wasmann et al. [40] | Perspective/Framework paper | To introduce computational audiology as a digital approach to hearing health. | Audiology workflows, digital datasets, device ecosystems. | Proposed conceptual framework with illustrative examples. |
| Wolfgang [18] | Expert commentary | To discuss AI and ML’s impact on hearing technology. | Industry and device innovation reports. | Commentary and professional opinion. |
| Zou [19] | Conceptual exploratory article | To explore AI use in HAs for improved functionality. | Literature, device innovations, market trends. | Conducted conceptual synthesis and exploratory analysis. |

Key: HA = hearing aid; CI = cochlear implant; ABR = auditory brainstem response; ML = machine learning; AI = artificial intelligence; HIC = high-income country; LMIC = low- and middle-income country; SMP = social media platforms; FATE = fairness, accountability, transparency, ethics; SSA = Sub-Saharan Africa.

**Evidence Table 2:** AI Focus and Populations

| **Authors** | **AI/ML Application Area** | **Specific Populations or Regions Focused On** | **General Findings Related to AI’s Impact** |
| --- | --- | --- | --- |
| Asiedu et al. [23] | Governance/ethics (cross-cutting) | Africa—multicountry; Global; LMICs; HICs; rural–urban; diverse demographic axes (ethnicity, language, gender, age) | AI can propagate Representation and Intersectional bias, with vulnerabilities shaped by colonial history and socioeconomic inequities. |
| Wilson et al. [13] | Clinical decision support; CI coding/outcomes; HA personalisation | LMICs; rural; children with hearing impairment; indigenous and refugee populations | AI offers promise in CIs, HAs, and diagnostics but exacerbates inequities through digital divide and underrepresentation of LMICs. |
| Koyama et al. [11] | Otological imaging/diagnosis; Clinical decision support | Global/unspecified | AI raises Measurement and Algorithmic bias due to data shifts, with major challenges in transparency and trust. |
| Lesica et al. [41] | Clinical decision support | HICs; LMICs | AI models built on HIC datasets risk poor generalisability for LMIC contexts due to dataset disparities. |
| Ferdous et al. [6] | Clinical decision support; Automated audiometry; Speech enhancement/noise reduction | LMICs; vulnerable populations | Smart Auditory Health Systems have transformative potential but face data scarcity, infrastructure gaps, and unresolved privacy/ethics challenges. |
| Hussain et al. [38] | Clinical decision support | US; marginalised populations (Black, Native American, Asian, Latinx) | AI health algorithms show Representation bias, underestimating risk in minorities and worsening inequities in treatment. |
| Ismail [16] | HA personalisation; Speech enhancement/noise reduction | Global/unspecified; HA users across age groups | AI enhances speech clarity and satisfaction in HAs but raises Algorithmic transparency and privacy concerns. |
| Fabry & Bhowmik [15] | HA personalisation | Global/unspecified | AI adoption in HAs is rapid, simulating human intelligence and enabling new applications. |
| Mwangi [17] | HA personalisation; Speech enhancement/noise reduction | Individuals with hearing impairment | AI-enhanced noise management improves outcomes but risks socioeconomic inequity in access and requires long-term studies. |
| Iliadou et al. [37] | Clinical decision support; HA personalisation | Global/unspecified; individuals with hearing loss | AI models predict HA outcomes but face Representation bias and limited generalisability for diverse populations. |
| Balling et al. [36] | HA personalisation | HICs; HA users (n=32,000 comparisons) | AI optimises HA settings from large-scale user data, reducing lab bias but still subject to selection effects. |
| Zhang et al. [20] | CI coding/outcomes; HA personalisation | Global/unspecified; CI and HA users | AI offers personalised and adaptive rehabilitation, improving quality of life for individuals with hearing loss. |
| Chinta et al. [42] | Clinical decision support | Global/unspecified; underrepresented groups (Black, darker-skinned, non-Western patients) | AI enhances efficiency but demonstrates Representation and Measurement bias when datasets exclude minority populations. |
| Moldovan et al. [3] | Clinical decision support | Global/unspecified; minorities; women; migrants; CYP; older adults | Algorithmic bias leads to diagnostic errors and stereotypes, worsened by underrepresentation and black-box models. |
| Pham [43] | Otological imaging/diagnosis; Clinical decision support | Global/unspecified | AI advances diagnostics but risks inequity due to homogenous datasets and unresolved privacy/legal issues. |
| Green [46] | Clinical decision support; Speech enhancement | Global/unspecified; individuals with speech impairment | AI supports advanced care in CSD, improving accessibility but requiring careful validation. |
| Singhal et al. [5] | Clinical decision support (social media data) | Younger, urban populations | AI use on SMPs risks Representation bias towards youth and urban users, with proprietary models limiting fairness and transparency. |
| Galiana et al. [44] | Clinical decision support; Pharmaceutical research | Global/unspecified | AI improves early detection and treatment but requires multidisciplinary oversight and algorithmic transparency. |
| Owoyemi et al. [24] | Clinical decision support; Otological imaging/diagnosis | Africa—multicountry; LMICs | AI in Africa faces Representation bias, cost barriers, and limited infrastructure, restricting applicability of imported models. |
| Janneker [45] | Clinical decision support | South Africa | AI bias reflects insufficiently diverse local datasets, embedding systemic inequalities and reducing equity. |
| Alaran et al. [25] | Clinical decision support; Governance/ethics (cross-cutting) | Africa—multicountry; LMICs; rural; vulnerable populations | AI can improve health outcomes but risks inequity without attention to equity, accountability, and socio-cultural factors. |
| Panch et al. [2] | Governance/ethics (cross-cutting) | Global/unspecified; diverse populations | AI risks Algorithmic and Deployment bias without diverse teams and context-specific fairness definitions. |
| Nazer et al. [4] | Clinical decision support | HICs; LMICs; minority and underrepresented groups | AI models exhibit Representation bias if trained on dominant populations; data diversification is essential. |
| Ugar [35] | Governance/ethics (cross-cutting) | Sub-Saharan Africa; global minorities (African Americans, Asians, Africans) | AI design that ignores African agency risks “technological colonialism” and cultural misfit. |
| Frosolini et al. [9] | Automated audiometry; Speech enhancement/noise reduction; Diagnostic imaging | Hearing-impaired populations | AI improves audiological diagnosis and rehabilitation but requires diverse datasets and privacy safeguards. |
| AlSamhori et al. [7] | Automated audiometry; HA personalisation | LMICs; noise-exposed workers; patients with hearing loss | AI strengthens diagnostic accuracy and rehabilitation but raises privacy/consent concerns and risks overfitting. |
| You et al. [14] | Automated audiometry; ABR classification; Speech enhancement/noise reduction; Otological imaging/diagnosis | Global/unspecified | AI offers innovations in otology but inherits bias from training datasets, requiring standardisation and validation. |
| Bur et al. [8] | Otological imaging/diagnosis; Clinical decision support | Global/unspecified | AI depends on large datasets, risking bias and privacy concerns from reliance on EHR data. |
| Celi et al. [1] | Governance/ethics (cross-cutting) | Global/unspecified | Bias arises across the AI lifecycle (data, modelling, deployment), producing inequitable outcomes. |
| Hagerty & Rubinov [39] | Governance/ethics (cross-cutting) | Global/unspecified | AI creates social and ethical risks, demanding governance and equity frameworks. |
| Wasmann et al. [40] | Computational audiology | HICs; hearing-impaired populations | Computational audiology can transform care but depends on high-quality data and interoperability. |
| Wolfgang [18] | HA personalisation | Global/unspecified; HA users | AI expands HA functionality, enabling noise reduction and better user experiences. |
| Zou [19] | HA personalisation | Global/unspecified; HA users | AI in HAs offers personalised performance but risks inequity if access is limited. |

Key: HA = hearing aid; CI = cochlear implant; ABR = auditory brainstem response; ML = machine learning; AI = artificial intelligence; HIC = high-income country; LMIC = low- and middle-income country; SMP = social media platforms; FATE = fairness, accountability, transparency, ethics; SSA = Sub-Saharan Africa.

**Evidence Table 3:** Biases, Ethical, Cultural & Linguistic Issues

| **Authors** | **Types of Bias Discussed** | **Ethical/Cultural/Linguistic Issues Mentioned** | **Consequences of Bias/Ethical Issues** | **Mitigation Strategies/Recommendations** |
| --- | --- | --- | --- | --- |
| Asiedu et al. [23] | Representation; Measurement; Algorithmic; Evaluation; Deployment; Intersectional | Data sovereignty; techno-colonial procurement; mistrust of foreign technologies; linguistic inequality; stigmatisation of gender non-conforming persons; ethno-religious disparities | Misclassification of thresholds; exclusion of minority groups; propagation of harmful stereotypes; reduced trust and uptake in LMICs | Contextual fairness criteria; participatory co-design; local dataset development; cautious use of pretrained models; African ownership and governance |
| Wilson et al. [13] | Representation | Digital divide; underrepresentation of LMICs, women, indigenous, and refugees; opacity of “black box” models | Errors in predictions for underrepresented groups; reduced clinical validity; exacerbation of digital inequities | Investment in LMIC digital infrastructure; inclusive dataset building; clinician–AI collaboration |
| Koyama et al. [11] | Representation; Algorithmic; Deployment | Legal liability; lack of transparency; privacy risks | Misdiagnosis; reduced reliability of AI-driven otological care; patient mistrust | Transparent model reporting; explainable AI; robust data governance |
| Lesica et al. [41] | Representation | Over-reliance on HIC datasets | Limited generalisability of hearing inference models; suboptimal clinical outcomes in LMICs | Creation of shared global hearing registries; inclusion of LMIC data |
| Ferdous et al. [6] | Algorithmic | Data privacy; inequitable access | Inequitable distribution of smart auditory health systems; exclusion of LMIC patients | Fairness-aware training; stronger privacy safeguards; equity-focused deployment |
| Hussain et al. [38] | Representation; Algorithmic; Deployment; Intersectional | Medical mistrust; racial stereotypes in health records; inequities in digital health literacy | Underdiagnosis in marginalised groups; postponed treatment; perpetuation of racial health inequities | Equity-centred algorithm design; community-engaged data collection; regular bias audits; regulatory oversight |
| Ismail [16] | Algorithmic | Privacy; user consent; transparency; human rights | Data misuse; inequitable access to AI-enabled hearing aids; loss of patient confidence | Ethical frameworks for assistive tech; strong data protection; transparent consent processes |
| Fabry & Bhowmik [15] | Representation | Need for relevant datasets; ethical considerations | Risk of biased learning if datasets are unrepresentative | Dataset diversification; fairness-aware training |
| Mwangi [17] | Not explicit (implied representation bias) | Data privacy; socio-economic disparities | Unequal access to advanced hearing aids; widening inequities | Ethics-focused product design; socioeconomic equity audits |
| Iliadou et al. [37] | Representation; Algorithmic | Black-box opacity; GDPR compliance; accountability | Unreliable hearing aid predictions; reduced patient trust | Transparent and interpretable modelling; culturally appropriate validation |
| Balling et al. [36] | Representation (selection bias) | Data anonymity and privacy | Risk of unrepresentative training data | Anonymised storage; scaling datasets |
| Zhang et al. [20] | Algorithmic (implied) | Consent; privacy | Suboptimal HA/CI integration; trust challenges | Multidisciplinary design teams; consent-driven development |
| Chinta et al. [42] | Representation; Measurement; Algorithmic; Evaluation | Informed consent; accountability; loss of trust | Misdiagnosis; inequitable resource allocation; systemic inequality | Fairness-aware algorithms; regulatory oversight; interpretable AI frameworks |
| Moldovan et al. [3] | Representation; Measurement; Algorithmic; Evaluation; Deployment | Gender inequities; migrant exclusion; transparency; fairness | Inaccurate diagnosis for women/ethnic minorities; perpetuation of stereotypes; erosion of trust | Diverse datasets; explainable AI; audit frameworks; Ubuntu ethics |
| Pham [43] | Representation; Algorithmic | Privacy; legal compliance; accountability | Exaggerated healthcare inequities; reduced generalisability | Diverse training data; regular audits; fairness constraints in models |
| Green [46] | Representation (implied) | Not explicit | Potential propagation of systemic inequities | Adoption of fairness-aware modelling |
| Singhal et al. [5] | Representation; Algorithmic | Fairness, Accountability, Transparency, Ethics (FATE); digital divide; data privacy | Bias toward younger/urban demographics; misinformation; privacy breaches | Fairness-aware design; auditability; intersectional fairness methods; explainability tools |
| Galiana et al. [44] | Representation | Ethical regulation; moral philosophy; liability; patient rights | Misclassification; inequitable treatment; discrimination | Multidisciplinary ethics integration; transparency in models; ongoing clinician training |
| Owoyemi et al. [24] | Representation; Algorithmic | Cultural appropriateness; privacy; accountability; transparency | Misrepresentation of African populations; inappropriate model transfer; limited adoption | African-led dataset creation; culturally aligned governance frameworks |
| Janneker [45] | Representation; Deployment | Historical/systemic inequalities; fairness; accountability | Inaccurate diagnosis in South Africa’s multiethnic population | Diverse local datasets; independent AI oversight; tailored infrastructure |
| Alaran et al. [25] | Representation; Algorithmic | Equity and access; socio-cultural impacts | Worsening inequities; poor generalisability; misinformation | Ethical frameworks; inclusive policy; equity-focused deployment |
| Panch et al. [2] | Algorithmic; Evaluation | Lack of fairness definition; opacity; clinician mistrust | Amplification of inequities; misclassification in underrepresented groups | Context-sensitive fairness criteria; diverse AI development teams |
| Nazer et al. [4] | Representation; Algorithmic; Evaluation | Trust; ethical oversight | Misclassification; poor performance on minorities | Data diversification; fairness-aware validation; continuous monitoring |
| Ugar [35] | Representation; Algorithmic; Deployment; Intersectional | Techno-colonialism; lack of African agency; cultural bias | Misrecognition of African patients; mistrust of foreign AI; poor uptake | African-designed AI; local data sovereignty; decolonised governance |
| Frosolini et al. [9] | Representation | Data privacy; ethical concerns | Misclassification; poor generalisation to LMICs | High-quality diversified datasets; robust privacy measures |
| AlSamhori et al. [7] | Algorithmic; Evaluation | Privacy; consent | Inaccurate audiometric classifications; reduced adoption | Multidisciplinary collaboration; ethical frameworks |
| You et al. [14] | Representation | Confidentiality; physician trust | Bias transfer into otological algorithms | Standardised datasets; rigorous validation; physician education |
| Bur et al. [8] | Representation | Privacy; confidentiality | Diminished utility of AI in ENT | Ethical frameworks; transparency and accountability |
| Celi et al. [1] | Representation; Measurement; Algorithmic; Deployment | LMIC underrepresentation; inequities in data access | Poor generalisability; exacerbated disparities | Diverse datasets; fairness-aware modelling; governance frameworks |
| Hagerty & Rubinov [39] | Representation; Deployment (generic) | Techno-colonialism; inequitable governance | Global reinforcement of inequities | Inclusive AI governance; global ethics frameworks |
| Wasmann et al. [40] | Representation (implied) | Digital divide | Unequal benefit from computational audiology | Equitable data sharing; LMIC accessibility |
| Wolfgang [18] | Not explicit | None | Optimistic framing obscures equity risks | Broader access strategies |
| Zou [19] | Algorithmic (optimisation bias) | None explicit | Limited HA benefits if unaffordable | Align HA innovation with affordability and accessibility |

Key: HA = hearing aid; CI = cochlear implant; ABR = auditory brainstem response; ML = machine learning; AI = artificial intelligence; HIC = high-income country; LMIC = low- and middle-income country; SMP = social media platforms; FATE = fairness, accountability, transparency, ethics; SSA = Sub-Saharan Africa.
